# Iron regulatory proteins 1 and 2 have opposing roles in regulating inflammation in bacterial orchitis

**DOI:** 10.1172/jci.insight.175845

**Published:** 2024-02-01

**Authors:** Niraj Ghatpande, Aileen Harrer, Bar Azoulay-Botzer, Noga Guttmann-Raviv, Sudhanshu Bhushan, Andreas Meinhardt, Esther G. Meyron-Holtz

**Affiliations:** 1 Faculty of Biotechnology and Food Engineering, Technion-Israel Institute of Technology, Technion City, Haifa, Israel.; 2 Institute of Anatomy and Cell Biology, Unit of Reproductive Biology, Justus-Liebig-University of Giessen, Giessen, Germany.

**Keywords:** Immunology, Reproductive biology, Bacterial infections, Macrophages, Neutrophils

## Abstract

Acute bacterial orchitis (AO) is a prevalent cause of intrascrotal inflammation, often resulting in sub- or infertility. A frequent cause eliciting AO is uropathogenic *Escherichia coli* (UPEC), a gram negative pathovar, characterized by the expression of various iron acquisition systems to survive in a low-iron environment. On the host side, iron is tightly regulated by iron regulatory proteins 1 and 2 (IRP1 and -2) and these factors are reported to play a role in testicular and immune cell function; however, their precise role remains unclear. Here, we showed in a mouse model of UPEC-induced orchitis that the absence of IRP1 results in less testicular damage and a reduced immune response. Compared with infected wild-type (WT) mice, testes of UPEC-infected *Irp1^–/–^* mice showed impaired ERK signaling. Conversely, IRP2 deletion led to a stronger inflammatory response. Notably, differences in immune cell infiltrations were observed among the different genotypes. In contrast with WT and *Irp2^–/–^* mice, no increase in monocytes and neutrophils was detected in testes of *Irp1^–/–^* mice upon UPEC infection. Interestingly, in *Irp1^–/–^* UPEC-infected testes, we observed an increase in a subpopulation of macrophages (F4/80^+^CD206^+^) associated with antiinflammatory and wound-healing activities compared with WT. These findings suggest that IRP1 deletion may protect against UPEC-induced inflammation by modulating ERK signaling and dampening the immune response.

## Abstract 2

Iron regulatory proteins 1 and 2 modulate the immune response and tissue damage during uropathogenic <em>Escherichia coli</em>-mediated orchitis.

## Graphical abstract

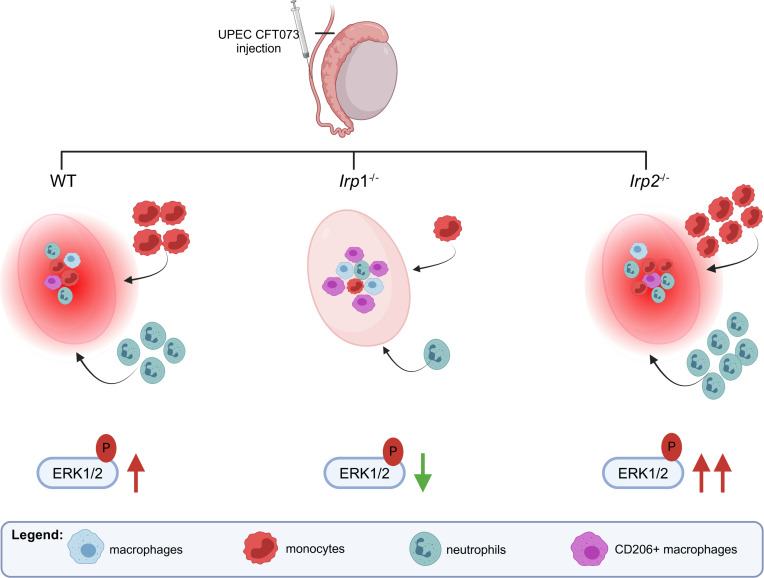

## Introduction

Acute orchitis (AO) is a prevalent cause of intrascrotal inflammation that results in approximately 600,000 medical visits per year in the United States alone and presents mostly as combined epididymo-orchitis (1). AO is predominantly attributed to uropathogenic *Escherichia coli* (UPEC) strains or sexually transmitted pathogens such as *Chlamydia trachomatis* (2, 3). This condition can result in infertility in males, and studies have shown that patients infected with UPEC have lower sperm counts even many months after successful antibiotic treatment (3–6).

Iron, an essential element for humans, plays an important role in spermatogenesis (7). Mammalian iron homeostasis is tightly regulated at both the systemic and cellular levels. Iron regulatory proteins 1 and 2 (IRP1 and IRP2, also known as ACO1 and IREB2, respectively) are crucial for maintaining cellular iron homeostasis (8, 9). This tight regulation ensures the balance of cellular iron levels (10). IRP2 deletion has been found to significantly alter iron metabolism, highlighting its critical role in cellular iron homeostasis, including the disruption of ferritin regulation (11, 12). Similarly, global deletion of IRP2 or systemic iron overload caused elevated ferritin levels in the testis of mice (13). In contrast, IRP1 deletion impairs iron metabolism only slightly and in a tissue-specific manner, as increased ferritin was mainly detected in kidney and brown fat pads, and dysregulated HIF2α led to transient splenomegaly and extramedullary hematopoiesis (14–16). In addition to their role in cellular iron homeostasis, recent studies have shown IRPs’ roles in immune regulation as well. For example, Bonadonna et al. showed that IRPs ensure that neutrophils have the iron they need to fight infection, and IRP deficiency leads to impaired neutrophil function and increased susceptibility to bacterial infections (17). Furthermore, ablation of IRP1 and IRP2 in macrophages leads to increased susceptibility to *Salmonella* infection, suggesting that these proteins are critical for limiting microbial iron acquisition and promoting host defense (18). However, the precise role of IRPs in the immune response remains poorly understood.

Iron is also an essential nutrient for the growth and survival of most bacterial species. During bacterial infection the host typically limits systemic iron availability to pathogens as a part of the immune response (19, 20). However, certain bacterial species, such as UPEC, have evolved specialized mechanisms to acquire iron from the host, potentially contributing to their virulence (21–23). Further studies have shown that limiting iron can reduce the bacterial burden (24). To circumvent the low iron availability, UPEC can chelate iron via siderophores from the host. Alternatively, UPEC can persist in cells within autophagosomes, where they can access free iron via ferritinophagy (25). In addition, in bladder infection, UPEC induced activation of Toll-like receptor 4 (TLR4), which is known to lead to the production of proinflammatory cytokines, including IL-6 and IL-1β (26, 27). In contrast, in rat testis, proinflammatory cytokines like TNF-α and IL-6 were not produced following UPEC infection (28). Furthermore, modified iron availability during bacterial infections influences the polarization of macrophages into classically activated (M1) or alternatively activated (M2) macrophages, depending on the location of infection (29, 30).

M2 macrophages, characterized, e.g., by the high constitutive production of immunosuppressive cytokines such as IL-10, play a relevant role in normal testicular homeostasis (31, 32). On the other hand, in AO, infiltrating monocytes and neutrophils are instrumental in causing the observed tissue damage (33–36). In this regard, it is completely unknown what role iron homeostasis plays in the development of AO and in the magnitude of the testicular immune response. Thus, in this study we aimed to investigate the role of iron homeostasis in UPEC-mediated AO in mice with targeted deletion of IRP1 or IRP2. The results emphasize the contribution of each IRP to the immune response following UPEC-induced AO, shedding light on their multifaceted functions beyond traditional iron regulation.

## Results

### IRP1 deficiency protects the testis against UPEC-induced inflammation.

**Figure 1:**
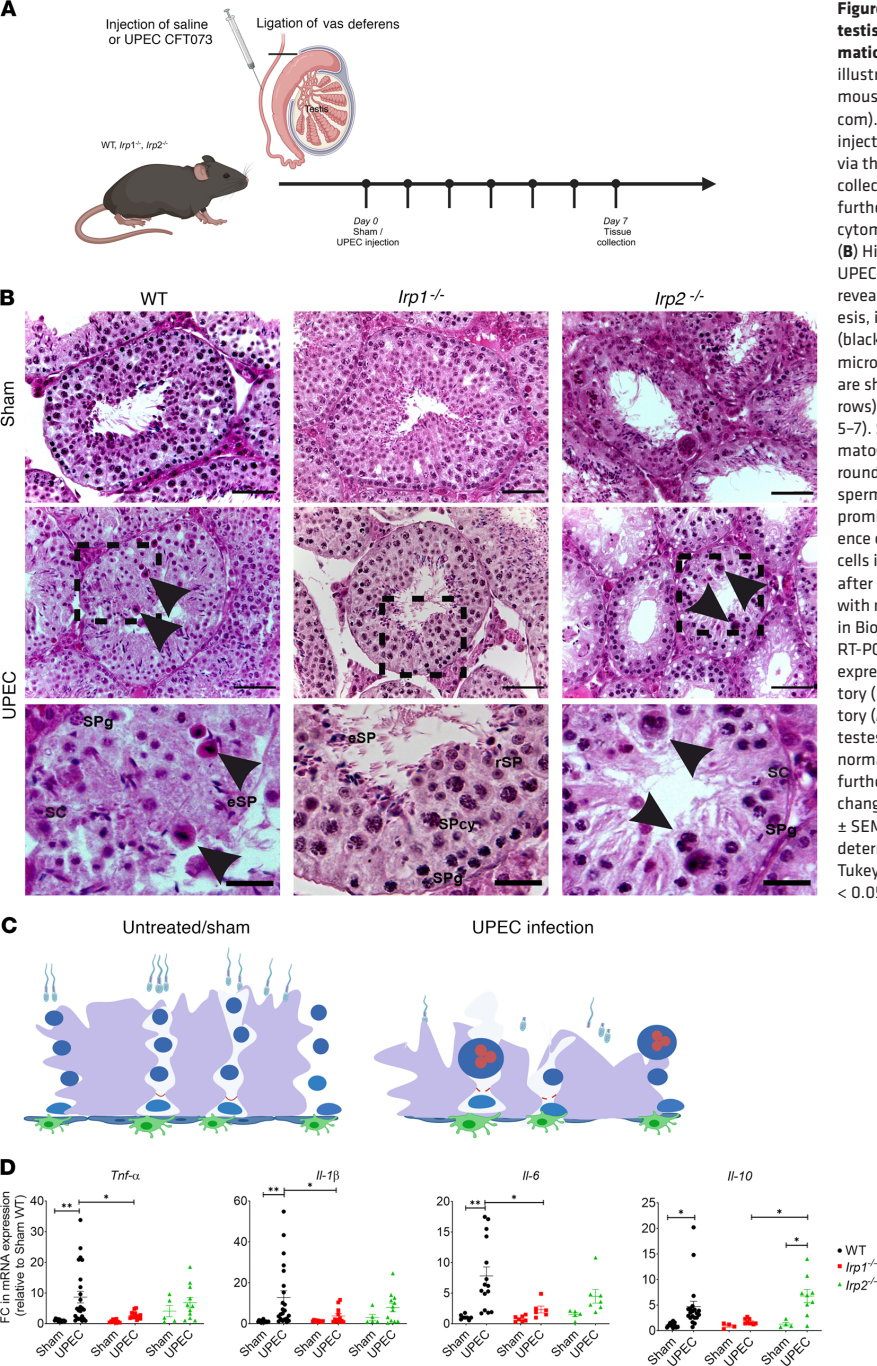
IRP1 deficiency protects the testis against UPEC-induced inflammation. (**A**) Experimental design illustrating the UPEC-induced orchitis mouse model (created in BioRender.com). WT, *Irp1^–/–^*, and *Irp2^–/–^* mice were injected with saline or UPEC CFT073 via the vas deferens. Organs were collected 7 days after infection for further analysis by histology, flow cytometry, and quantitative RT-PCR. (**B**) Histopathological analysis of UPEC-infected WT and *Irp2^–/–^* testes revealed impairment of spermatogenesis, including multinucleated cells (black arrowheads). Representative micrographs of H&E-stained testes are shown. Scale bars: 50 μm (top 2 rows) and 20 μm (bottom row) (*n* = 5–7). SC, Sertoli cells; SPg, spermatogonia; SPcy, spermatocytes; rSP, round spermatids; eSP, elongated spermatids. (**C**) The scheme illustrates prominent changes such as the presence of multinucleated giant germ cells in the seminiferous epithelium after UPEC infection in comparison with normal/sham conditions (created in BioRender.com). (**D**) Quantitative RT-PCR analysis demonstrated altered expression levels of key proinflammatory (*Tnf*, *Il-1β*, *Il-6*) and antiinflammatory (*Il*-*10*) cytokines in UPEC-infected testes. Relative mRNA levels were normalized to *Rplp0* or *18S* RNA and further to sham WT (*n* = 6–15). FC, fold change. Data are presented as mean ± SEM. Statistical significance was determined using 2-way ANOVA with Tukey’s multiple-comparison test. *P < 0.05; **P < 0.01.

To examine whether inflammatory effects of UPEC infection were exacerbated or attenuated in *Irp1^–/–^* and *Irp2^–/–^* mice compared with wild-type (WT) mice, UPEC was injected into both vasa deferentia of mice close to the epididymides and effects were monitored after 7 days in the testis (Figure 1A). Testicular weight was similar in sham and UPEC-infected mice of all 3 genotypes (Supplemental Figure 1E; supplemental material available online with this article; https://doi.org/10.1172/jci.insight.175845DS1). Yet, H&E-stained testicular sections revealed notable changes in testicular morphology in WT and *Irp2^–/–^* infected mice (Figure 1B). Higher-magnification images point to strong changes in infected WT and *Irp2^–/–^* testes that are accompanied by loss of germ cells, particularly elongated spermatids (eSPs). Furthermore, frequently multinucleated giant cells are seen. These cells represent degenerating germ cells (black arrowheads) that evolve as a consequence of fusion of the cellular content of conjoined germ cells (Figure 1C). The thin cytoplasmic bridges that normally remain after incomplete cytokinesis, widen abnormally, and allow fusion of the cellular contents (37). Surprisingly, *Irp1^–/–^* mice still exhibit the presence of eSPs and display few to no formation of multinucleated giant cells following UPEC infection, even though bacterial loads in *Irp1^–/–^* mice were comparable to those in WT and *Irp2^–/–^* mice (Supplemental Figure 1, F and G). Hypospermatogenesis, which represents a testicular impairment when all germ cell types are present but all or some populations are decreased in number, was also evident in sham *Irp1^–/–^* testis (Figure 1B).

To gain further understanding of the observed decrease in testicular damage in UPEC-infected *Irp1^–/–^* mice, we analyzed possible changes in immune response by quantifying the mRNA expression levels of key proinflammatory (*Tnf*, *Il-6*, and *Il-1β*) and antiinflammatory (*Il-10*) cytokines using quantitative RT-PCR, where a significant increase in cytokine transcript levels in UPEC-infected WT and *Irp2^–/–^* testes was noted compared with sham-infected testes (Figure 1D).

### Iron status remains unchanged after UPEC infection.

As UPEC infection can mediate significant changes in the iron status (24), we examined cellular iron status in the testis 7 days after UPEC infection and focused on proteins regulated by IRPs. No significant changes in the expression levels of the ferritin subunits (FtH and FtL) and transferrin receptor 1 (*Tfr1*) were observed in response to the infection across different genotypes at both the protein and mRNA level, respectively (Supplemental Figure 2, A–D). Further analysis using immunofluorescent staining on WT mouse testis revealed that ferritin was predominantly localized within the interstitium, primarily in macrophages (Supplemental Figure 2E).

### The ERK signaling pathway is impaired in Irp1^–/–^ testes.

As the absence of an inflammatory response in *Irp1^–/–^* mice could not be explained by differences in the iron status, we asked whether our observation could be explained by a modified activation of the proinflammatory MAPK and NF-κB pathways that are known to play a role in UPEC-elicited testicular inflammation (28, 38, 39).

**Figure 2:**
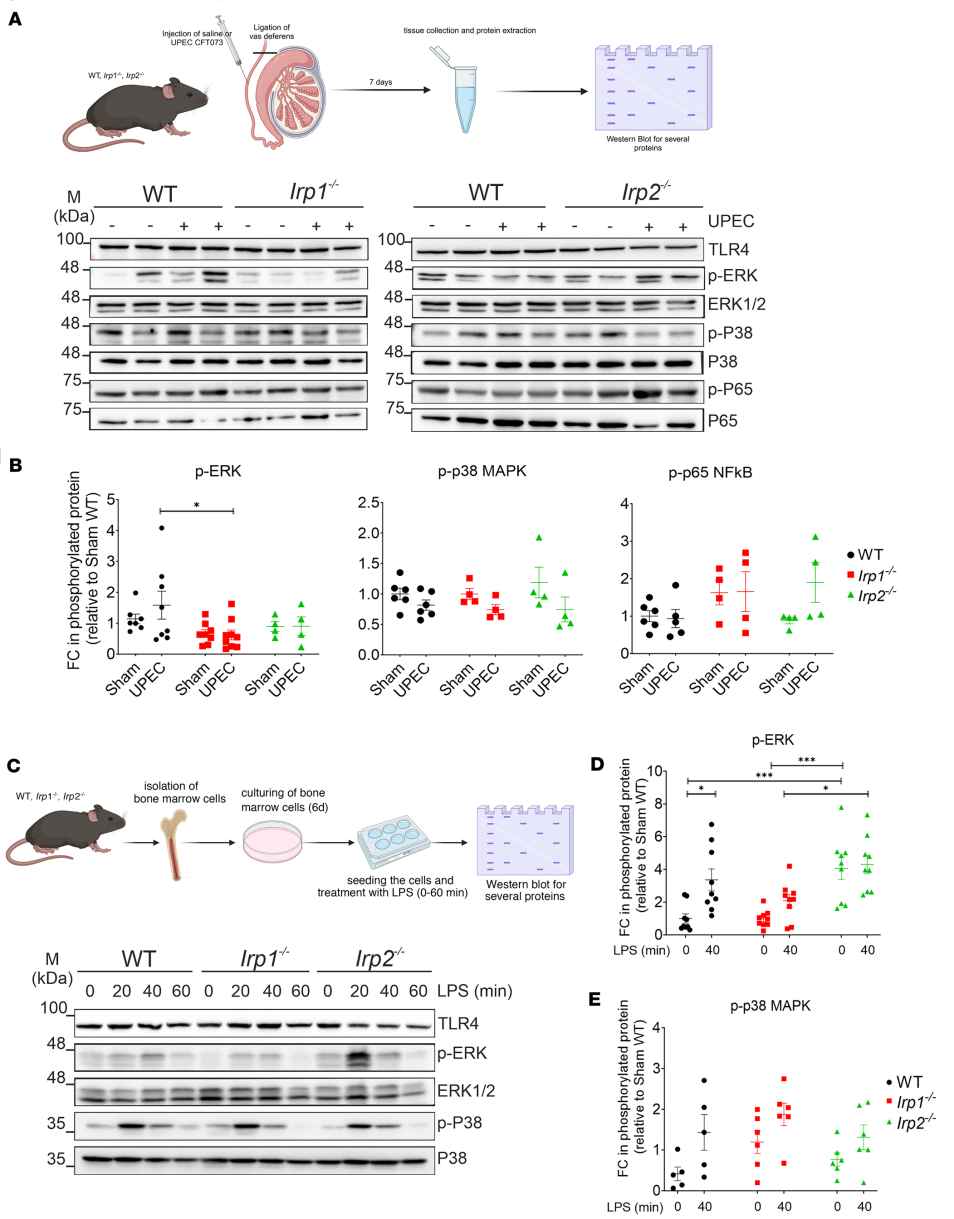
Impairment of the ERK signaling pathway in *Irp1^–/–^* testes. (**A**–**E**) Protein expression changes of various signaling proteins were analyzed by Western blotting. (**A**) The experimental workflow is shown (created in BioRender.com). Levels of targeted proteins (TLR4, p-ERK, total ERK, p-p38, p38, p-p65, and p65) are shown. (**B**) The band intensities of p-ERK, p-p38, and p-p65 were quantified using ImageJ and normalized to the corresponding loading control (*n* = 6). FC, fold change. (**C**) BMDMs from WT, *Irp1^–/–^*, and *Irp2^–/–^* mice were isolated and treated with 200 ng/mL LPS for different time points (20, 40, and 60 minutes; see Supplemental Figure 4) under 6% O_2_ at 37°C. Extracted proteins from isolated BMDMs were analyzed by Western blotting. Representative blots from 3 independent experiments are shown (each experiment is a pool of 2 to 3 animals). (**D** and **E**) Quantitative analysis was performed as described above. Data are presented as mean ± SEM. Statistical significance was determined using 2-way ANOVA with Tukey’s multiple-comparison test. *P < 0.05; ***P < 0.001.

Seven days after infection, levels of TLR4 and phosphorylated p65 (p-p65; NF-κB pathway) remained unchanged among all genotypes. In contrast, levels of p-ERK were significantly lower in response to UPEC infection in *Irp1^–/–^* testis compared with WT organs, whereas p-p38 remained unchanged (Figure 2, A and B). Activation of the ERK signaling pathway is generally rapid and transient after infection and inflammation (within minutes) (40). Therefore, 7 days after infection activation of ERK may not be detectable. Hence, we tested activation of ERK signaling response in bone marrow–derived macrophages (BMDMs) after short-term stimulation with lipopolysaccharide (LPS). Indeed, p-ERK levels were increased in WT and *Irp2^–/–^* BMDMs only moderately, compared with those seen in BMDMs from *Irp1^–/–^* mice (Figure 2, C and D, and Supplemental Figure 4). Basal levels of p-ERK in BMDMs of *Irp2^–/–^* mice were significantly higher than in WT and *Irp1^–/–^* mice. No significant differences in phosphorylation of p38 upon LPS treatment were observed across all genotypes (Figure 2E and Supplemental Figure 4).

### Differential immune cell infiltration following UPEC infection in Irp1^–/–^ and Irp2^–/–^ testes.

We speculated that the reduced p-ERK signaling observed in infected *Irp1^–/–^* mice could be accompanied by changes in leukocytic infiltration in the testis during UPEC infection (41). Flow cytometry analysis showed no significant differences in total leukocytes (CD45^+^ cells) in naive mice among genotypes (Supplemental Figure 1, A and B). Similarly, no significant differences in leukocytes were observed in the blood across genotypes (Supplemental Figure 1D). However, we found a slight reduction in the proportion of macrophages (F4/80^+^CD11b^+^) in total CD45^+^ leukocytes in testes from naive *Irp2^–/–^* mice as compared with testes from naive WT and *Irp1^–/–^* mice (Supplemental Figure 1C). Circulating macrophages (F4/80^+^CD11b^+^) in the blood did not exhibit such differences (Supplemental Figure 1D).

**Figure 3:**
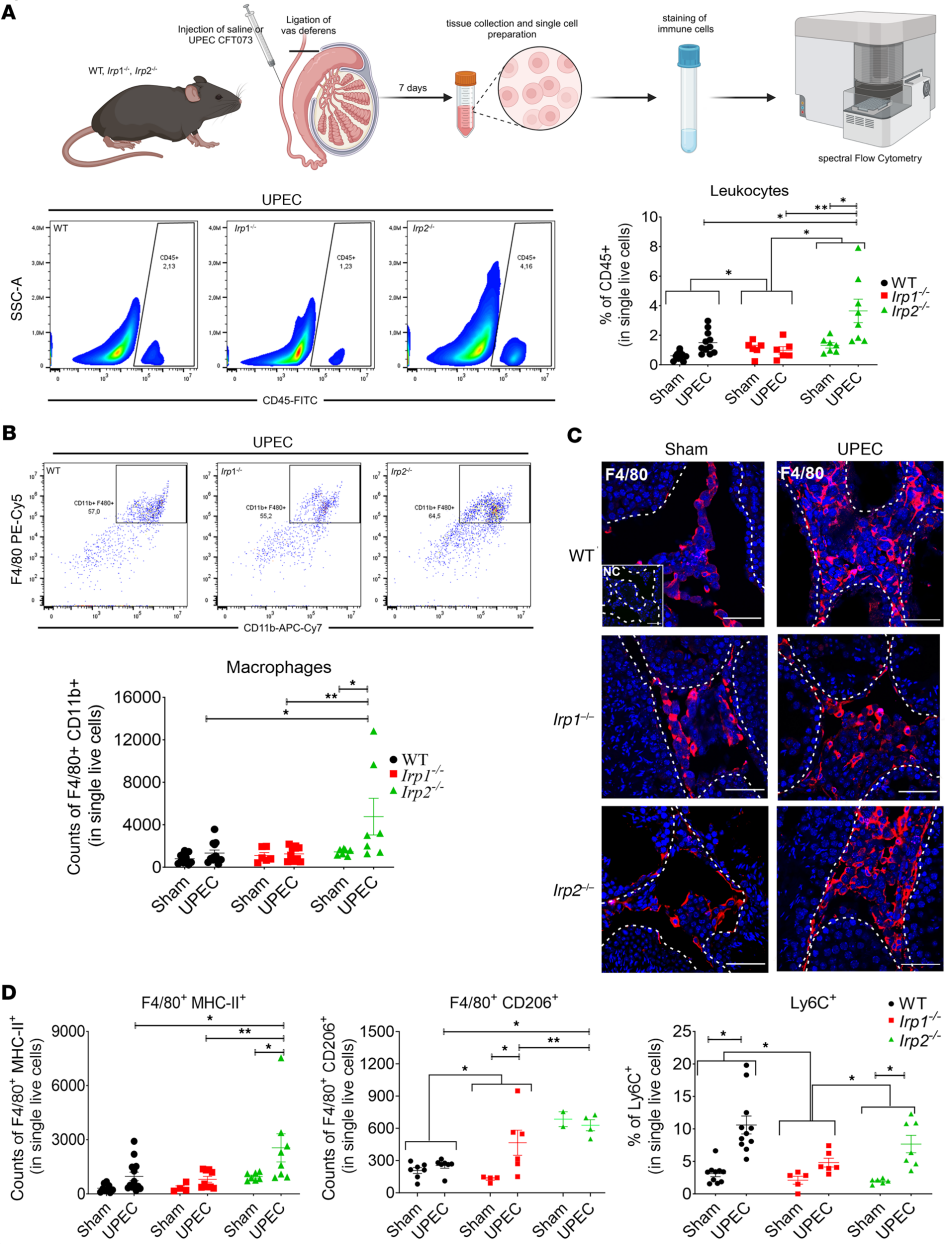
UPEC-induced leukocytic infiltration is lower in *Irp1^–/–^* testes. (**A**) The scheme shows the experimental workflow for flow cytometry analysis (created in BioRender.com). Representative flow cytometry plots and corresponding dot plots depict the total leukocyte population (CD45^+^, in 2 × 10^5^ single live cells) after UPEC infection. (**B** and **C**) Detailed analysis of total macrophages (CD11b^+^F4/80^+^ in 2 × 10^5^ single live cells) by flow cytometry. (**C**) Immunofluorescence of macrophages (F4/80^+^, red) shows localization in the testes of all genotypes. Data were obtained from 6–9 mice per group. Scale bars: 50 μm. (**D**) Subpopulations of macrophages (counts of F4/80^+^MHC-II^+^ and F4/80^+^CD206^+^, in 2 × 10^5^ single live cells) and monocytes (percentage of Ly6C^+^ cells in 2 × 10^5^ single live cells) in testes of different mouse genotypes by flow cytometry. Data are presented as mean ± SEM (*n* = 2–9). Statistical significance was determined using 2-way ANOVA with Tukey’s multiple-comparison test. *P < 0.05; **P < 0.01.

As seen previously during UPEC infection, we observed an increase in total (CD45^+^) leukocytes in WT testes compared with sham and found a similar pattern in testes of *Irp2^–/–^* mice (Figure 3A). However, in UPEC-infected *Irp1^–/–^* testes, no changes in the percentage of CD45^+^ cells were seen compared to their sham control (Figure 3A). Furthermore, the effect of UPEC infection on leukocytes was compared between genotypes. Here, leukocytic infiltration was significantly lower in testes from *Irp1^–/–^* mice compared with testes from WT and *Irp2^–/–^* mice.

Further flow cytometry analysis revealed no increase in total macrophage numbers (F4/80^+^CD11b^+^) in UPEC-infected *Irp1^–/–^* testes, while in *Irp2^–/–^* mice a strong increase was observed. (Figure 3B). Immunofluorescence analyses supported the observation from flow cytometry, showing increased numbers of F4/80^+^ cells within the interstitium of WT and *Irp2^–/–^* testes after UPEC infection (Figure 3C) compared with the sham controls, whereas no increase was seen in *Irp1^–/–^* testes.

The MHC-II^+^ macrophage subpopulation showed a similar elevation in UPEC-infected WT and *Irp2^–/–^* testes but not in *Irp1^–/–^* testes (Figure 3D) compared with the corresponding sham controls. However, the subpopulation of CD206^+^ macrophages was elevated exclusively in UPEC-infected *Irp1^–/–^* testes compared with sham *Irp1^–/–^* testes (Figure 3D). Moreover, this increase in CD206^+^ macrophages after UPEC infection in testes from *Irp1^–/–^* mice was significantly different from the UPEC effect on WT. This is important, as CD206^+^ macrophages play a crucial role in immunoregulation and in the resolution of tissue inflammation in the testis and other organs (42). Concomitant with macrophages, monocytes (Ly6C^+^ cells) also showed a significant increase only in WT and *Irp2^–/–^* testes after UPEC infection (Figure 3D), whereas UPEC-infected *Irp1^–/–^* testes showed no changes in the numbers of infiltrated monocytes (Ly6C^+^ cells) compared to corresponding sham control. In addition, comparing between genotypes, Ly6C^+^ monocyte infiltration after UPEC infection was significantly higher in WT and *Irp2^–/–^* compared with *Irp1^–/–^* mice.

**Figure 4:**
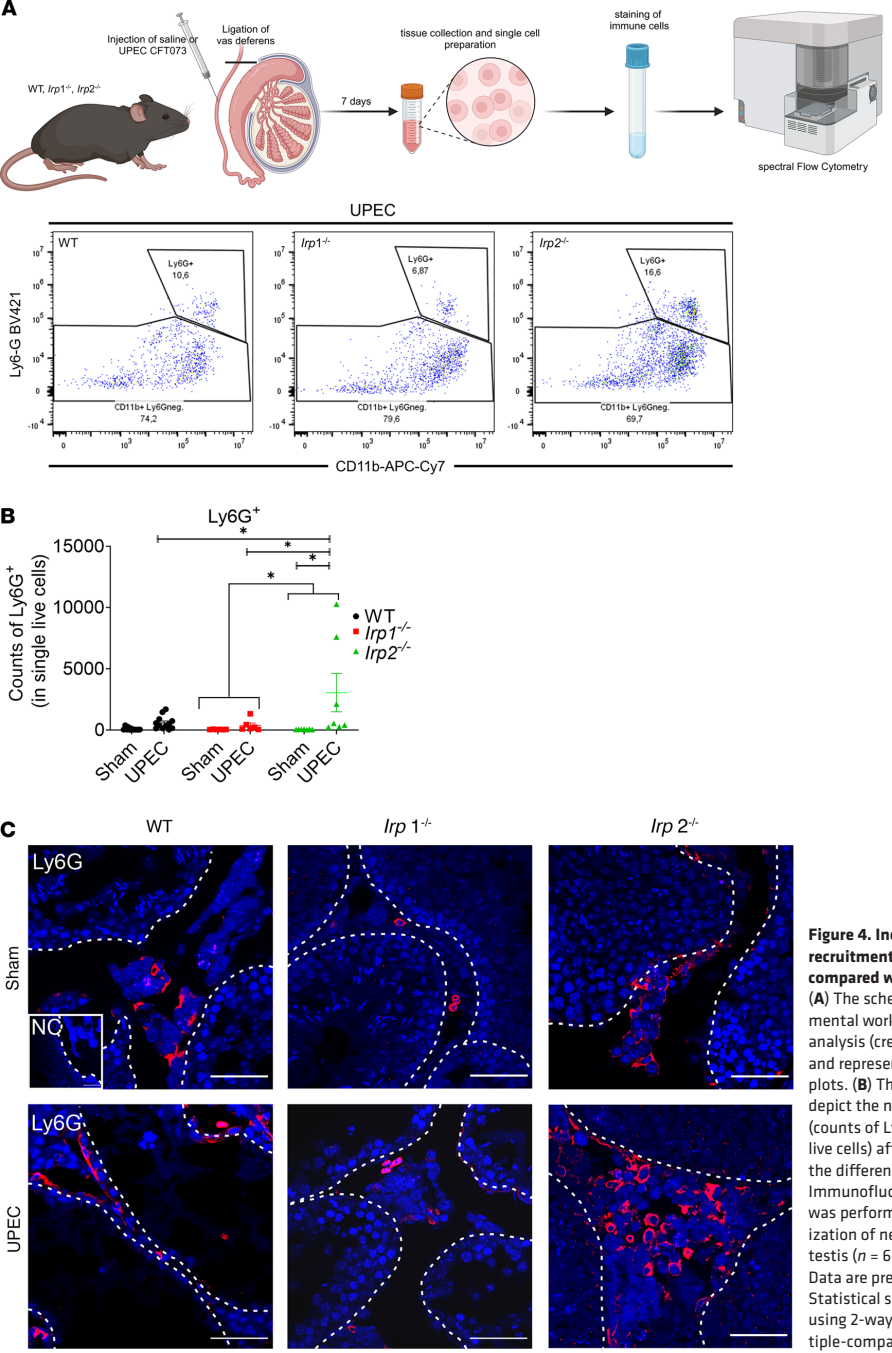
Increased neutrophil recruitment in infected *Irp2^–/–^* testes compared with WT and *Irp1^–/–^* mice. (**A**) The scheme shows the experimental workflow for flow cytometry analysis (created in BioRender.com) and representative flow cytometry plots. (**B**) The corresponding dot plots depict the neutrophil population (counts of Ly6G^+^ cells in 2 × 10^5^ single live cells) after UPEC infection across the different genotypes (*n* = 6–9). (**C**) Immunofluorescent staining of Ly6G was performed to visualize the localization of neutrophils in UPEC-infected testis (*n* = 6–9). Scale bars: 50 μm. Data are presented as mean ± SEM. Statistical significance was determined using 2-way ANOVA with Tukey’s multiple-comparison test. *P < 0.05.

Neutrophil numbers (Ly6G^+^) in the *Irp2^–/–^* testis were also elevated after UPEC infection compared with the respective sham control. This increase in neutrophil infiltration was also significantly different when comparing *Irp2^–/–^* with *Irp1^–/–^* mice (Figure 4, A and B). Immunofluorescence analysis of neutrophils (Ly6G^+^) showed that the infiltration following UPEC infection was confined to the interstitium of *Irp2^–/–^* testes, in accordance with the flow cytometry data (Figure 4C). No differences in T cell (CD3^+^) numbers across genotypes were evident. B cell (CD19^+^) numbers decreased in WT-infected testis compared with the respective sham control (Supplemental Figure 3, C and D).

**Figure 5:**
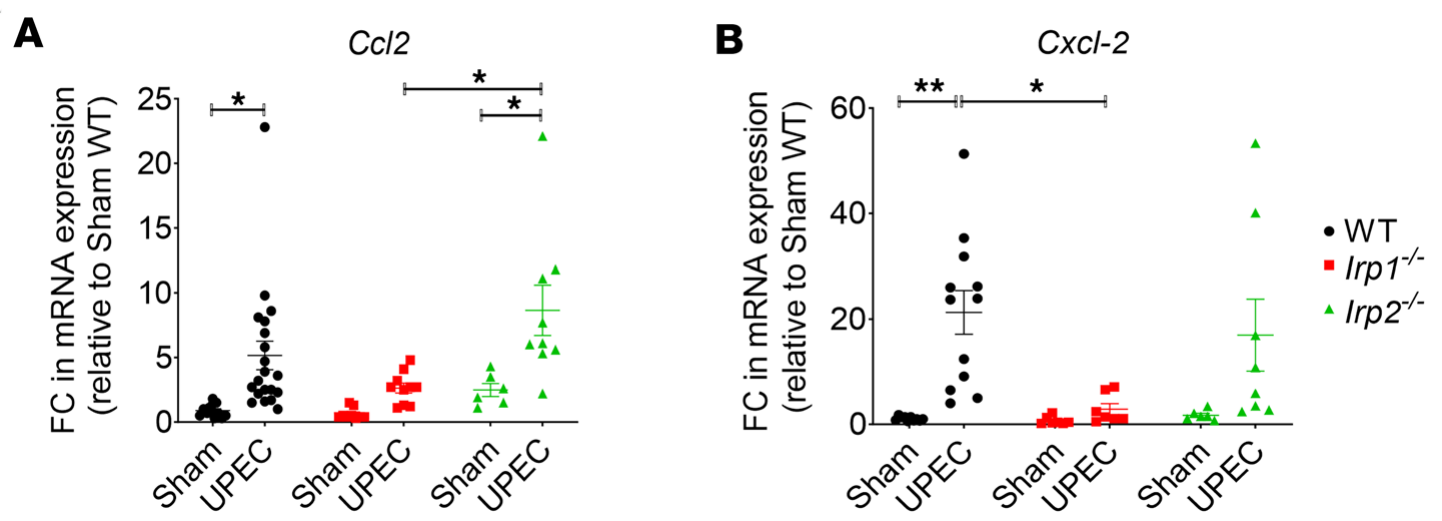
Lower chemokine levels in infected *Irp1^–/–^* testes compared with WT and *Irp1^–/–^*. (**A** and **B**) Expression of chemokine levels for the recruitment of monocytes/macrophages (*Ccl2*) and neutrophils (*Cxcl*2) were evaluated by qRT-PCR. FC, fold change. Data were obtained from 6–15 mice per group and are presented as mean ± SEM. Statistical significance was determined using 2-way ANOVA with Tukey’s multiple-comparison test. *P < 0.05; **P < 0.01.

Further analysis of chemokine expression related to monocyte, macrophage (*Ccl2*), and neutrophil (*Cxcl2*) recruitment revealed lower expression of *Ccl2* and *Cxcl2* in testes from UPEC-infected *Irp1^–/–^* mice compared with testes from UPEC-infected WT and *Irp2^–/–^* mice, both of which were elevated (Figure 5, A and B) compared with their sham controls.

## Discussion

The multifaceted roles of IRP1 and IRP2 have been extensively investigated across various studies, shedding light on their significance in cellular iron regulation and broader functionalities. In mouse models, local or global deletion of IRPs has revealed their diverse roles in embryonic development, immune responses mediated by macrophages, granulopoiesis, and T cell expansion (43). Both proteins exert tissue-specific effects, e.g., due to variations in their expression and distinct response to tissue oxygen levels (44).

Under normal conditions, IRP1 primarily serves as a cytosolic aconitase due to its stable 4Fe-4S cluster, while IRP2 dominates the regulation of cellular iron levels. Individual deletion of IRP1 and IRP2 has demonstrated contrasting outcomes in different tissues. For example, the deletion of IRP1 leads to polycythemia due to elevated HIF2α synthesis in the kidney, while this is not observed in *Irp2^–/–^* mice (14). Conversely, *Irp2^–/–^* mice exhibit a refractory anemia and neurodegeneration, a condition not reported in *Irp1^–/–^* mice (11, 12, 16). IRP2 deletion disrupts ferritin regulation, resulting in higher ferritin levels in all organs tested, including the testis (13, 16). These findings collectively highlight the distinct roles of IRP1 and IRP2 in maintaining cellular iron homeostasis.

The present study provides insights into the role of the iron regulatory proteins IRP1 and IRP2 in modulating the immune response and tissue damage elicited by UPEC-mediated orchitis. In the *Irp1^–/–^* testis, the dampened immune response was supported by a decreased ERK1/2 signaling, with subsequent lower expression of pro- and antiinflammatory cytokines (*Tnf*, *Il-1β*, and *Il*-6). Many testicular cells express TLRs and pattern-recognition receptors, indicating that they are capable of mounting an inflammatory response (28, 45). However, limited in vitro studies suggest that Sertoli cells, Leydig cells, peritubular cells, and macrophages can activate MAP kinase signaling pathways, including ERK1/2. Upon stimulation with various inflammatory stimuli, such as LPS, galectin, high-mobility group box protein 1 (HMGB1), and UPEC, these cells subsequently secrete inflammatory cytokines. In particular, testicular macrophages showed a robust inflammatory response (28, 32, 46–48). Based on these reports and our results presented here, we suggest that activated resident macrophages along with infiltrating immune cells, mainly monocytes and neutrophils, likely play a crucial role in activating the MAP kinase pathway and subsequent cytokine release in the testis. However, we also anticipate that other testicular cells may contribute to the inflammatory response, albeit to a lesser extent.

Based on the critical role of ERK1/2 signaling in immune cell activation and tissue inflammation (49, 50), impaired ERK1/2 activation in testes and BMDMs from *Irp1^–/–^* mice suggests a potential regulatory mechanism by which IRP1 interferes with signaling pathways during UPEC infection. This is supported by the notion that ERK interacts with both isoforms of aconitase (ACO1 and ACO2) and that inhibition of mitochondrial aconitase’s interaction with ERK1/2 reduced ERK signaling and the phosphorylation of downstream targets such as p90 ribosomal S6 kinase (RSK) (41).

Previous studies have highlighted the involvement of IRPs in modulating inflammatory responses and immune cell function (17, 18, 51). As an example, global disruption of IRPs impairs the development and differentiation of neutrophils in bone marrow of adult mice (17). Moreover, IRPs protect the host from *Salmonella* infection by reducing the intracellular proliferation of bacteria through control of iron availability (18). In our study, deletion of IRP2 in naive animals led to a reduction in F4/80^+^CD11b^+^ testicular macrophage numbers without affecting the circulating immune cells. This was accompanied by a massive infiltration of monocytes (Ly6C^+^), macrophages (MHC-II^+^), and neutrophils (Ly6G^+^) in UPEC-infected *Irp2^–/–^* testes, all of which are known for their potential to cause tissue damage through the production of reactive oxygen species (ROS) (52) and proinflammatory cytokines (53). We thus suggest that the noted immune cell recruitment is an instrumental part of the strong inflammatory response and tissue damage observed in *Irp2^–/–^* testes.

In addition to immune cell population dynamics, we investigated the expression of chemokines associated with monocyte (*Ccl2*), macrophage (*Ccl2*), and neutrophil (*Cxcl2*) recruitment. Our quantitative RT-PCR analysis revealed lower expression levels of *Ccl2* and *Cxcl2* in UPEC-infected *Irp1^–/–^* testes compared with highly elevated levels in infected WT and *Irp2^–/–^* testes (Figure 5). These chemokines play a critical role in the recruitment of immune cells to the site of infection and thus contribute to the inflammatory response. The consistent observation of a lack of significant leukocytic infiltration in the testes of UPEC-infected *Irp1^–/–^* mice, with the notable exception of the CD206^+^ resident macrophage subpopulation, suggests a role for IRP1 in the recruitment of leukocytes to the testis during infection. These CD206^+^ macrophages are known as immunoregulatory macrophages and play a crucial role in the resolution of tissue inflammation and are established players in the immunoregulation of the testis (4, 42, 54). Their functions extend beyond immune surveillance, as they actively contribute to the regulation of local inflammatory processes and the maintenance of tissue homeostasis. The reduced inflammation and absence of morphological changes seen in UPEC-infected *Irp1^–/–^* testes could thus be based on 2 mechanisms, i.e., the lack of infiltrating proinflammatory cells such as neutrophils and monocytes concomitant with an increase in CD206^+^ immunoregulatory tissue-preserving macrophages. In contrast to the response of *Irp2^–/–^* mice to UPEC infection, where an exacerbated activation of the ERK pathway was seen in conjunction with higher tissue damage, in *Irp1^–/–^* mice, the immune response seems to be dominated by the reduced ERK signaling.

In summary, our study provides insights into the role of IRP1 and IRP2 in modulating the immune response and tissue damage during UPEC-mediated orchitis. These findings suggest the potential of targeting IRPs as a therapeutic approach to mitigate testicular damage in bacterial infections. The comprehensive analysis of p-ERK levels, chemokine expression, and immune cell dynamics contribute to our understanding of the intricate interplay between iron metabolism, immune signaling, and tissue homeostasis in testicular inflammation. However, the detailed mechanism underlying why IRP1 and IRP2, which have been demonstrated to similarly regulate iron homeostasis, have opposing roles in regulating inflammation in UPEC-induced orchitis remains elusive.

## Methods

### Sex as a biological variable.

Only male mice were used in this study, as this study is about the male reproductive system.

### Mice.

The C57BL/6J mouse strain (Jackson Laboratory) was used for all experimental models. *Irp1^–/–^* (*Aco1^tm1Roua^*) and *Irp2^–/–^* (*Ireb2^tm1Roua^*) mouse strains were provided by Tracey Rouault (Molecular Medicine Program, National Institute of Child Health and Human Development, NIH, Bethesda, Maryland, USA). All male mice used in the experiments were age-matched (adult 10 to 12 weeks of age) and were randomly divided into each experimental group.

### Induction of bacterial orchitis.

The UPEC strain CFT073 was obtained from ATCC and propagated following established protocols (28, 55). To induce an ascending canalicular infection, we performed bilateral ligation of the vasa deferentia followed by intravasal injection of UPEC (1 × 10^5^ colony forming units [CFUs] in 10 μL sterile 0.9% NaCl) near the cauda using a Hamilton syringe. The control group (referred to as sham mice) underwent the same surgical procedure but received an intravasal injection of 10 μL sterile 0.9% NaCl. Mice were euthanized on day 7 after infection through isoflurane narcosis followed by cervical dislocation. The selection of this time point was based on a previously published report (55).

### Histology.

The testes were carefully dissected and immediately immersed in Bouin’s fixative (Sigma-Aldrich, HT10132-1L) for 6 hours. Following fixation, the tissue was embedded in paraffin and cut into 5-μm sections using a microtome and subsequently stained with H&E.

### Determination of CFU.

Testicular samples from both sham and UPEC-infected mice at 7 days after infection were homogenized in sterile ice-cold PBS (*n* = 4–7 per group). Subsequently, tissue homogenates were subjected to 10-fold serial dilutions. One hundred microliters of each dilution was streaked onto lysogeny broth (LB) agar plates. The plates were inverted and incubated at 37°C overnight. After incubation, CFUs on each plate were counted, and the values were normalized to tissue weight (per gram of used tissue).

### Immunofluorescence.

For immunofluorescent staining of immune cell types in testicular cryosections, 10-μm sections were fixed in methanol at –20°C for 20 minutes. Following fixation, the sections were washed 3 times and permeabilized with 0.5% Triton X-100 in 1× Tris-buffered saline (TBS) for 40 minutes. Subsequently, blocking was performed with 10% goat serum in 1× TBS for 1 hour. Sections were incubated with an anti-F4/80 antibody (1:100 dilution; Bio-Rad, catalog MCA497G) or an anti-Ly6G antibody (1:150 dilution; Abcam, catalog ab25377) overnight at 4°C. After incubation, the sections were washed 3 times and incubated with secondary antibodies (goat anti–rat IgG [H+L] cross-adsorbed secondary antibody Alexa Fluor 546, Invitrogen, catalog A-11081) for 1 hour at room temperature. Finally, the sections were mounted and images analyzed on a Zeiss LSM 710 confocal microscope. Adjustments to brightness and contrast on entire images were made using ImageJ (NIH). A total of 3 mice per genotype and treatment were included in the analysis.

### Preparation of single-cell suspension for flow cytometry.

Testes were collected into 2 mL tubes with Dulbecco’s modified Eagle medium (DMEM) (Thermo Fisher Scientific, 41965039) containing 10% fetal bovine serum (FBS) (Thermo Fisher Scientific, 10270106), 1% glutamine (Thermo Fisher Scientific, 25030024), and 1% penicillin-streptomycin (Thermo Fisher Scientific, 15140122) (“complete DMEM”). For mechanical separation, tissues were minced with scissors. Next, the tissues were digested in complete DMEM containing 1 mg/mL collagenase D (Sigma-Aldrich, 11088866001) and 1 μL DNase (Sigma-Aldrich, 4716728001) for 30 minutes at 37°C under shaking conditions. The cell suspension was further processed by aspiration through a 20-G needle and a 70-μm cell strainer (Sigma-Aldrich, CLS431751-50EA). The cell suspension was treated with red blood cell (RBC) lysis buffer (Biological Industries, 01-888-1B) to remove RBCs.

Blood was collected by cardiac puncture and transferred to an anticoagulant blood collection tube (BD, 368841). The blood was then subjected to 2 to 3 rounds of RBC lysis each for 5 minutes at room temperature until no RBCs were visible. The lysis reaction was stopped after each step by adding excess cold PBS, followed by centrifugation at 450*g* for 5 minutes at 4°C.

After preparing a single-cell suspension, cells were counted and 1 × 10^6^ cells/100 μL were used for staining. For live/dead estimation, the cells were first stained with Zombie Yellow (15 minutes at room temperature, in the dark) and then washed with flow buffer (0.5 M EDTA pH 8.0, 2.5% BSA in PBS). The cells were then blocked with anti–mouse CD16/CD32 antibodies for 10 minutes at 4°C and then incubated with the proper antibodies (see Tables 1 and 2) for 30 minutes at 4°C.

After the incubation, cells were washed with flow buffer and fixed with 1% paraformaldehyde (Barnaor, BN15710) for 15 minutes at 4°C. After washing with cold PBS, the cells were resuspended in flow buffer for analysis on the Cytec Aurora Spectral Flow cytometer, which was made accessible by the Technion Life Sciences and Engineering Infrastructure Centre in Haifa, Israel. The data were analyzed with FlowJo software version 10.8.1, according to gating strategy shown in Supplemental Figure 3, A and B.

### SDS-PAGE and Western blotting.

Tissue samples were immediately collected and snap-frozen at –80°C for subsequent analysis. Approximately 10 mg of testicular tissue was homogenized in RIPA buffer (50 mM Tris-HCl pH 7.4, 150 mM NaCl, 0.5% deoxycholate, 0.1% SDS, 1% NP-40, 0.1 M AEBSF, 1 mM DTT) supplemented with phosphatase inhibitor (Roche, 4906845001) and protease inhibitor cocktail (Roche, 11836170001) followed by incubation on ice for 30 minutes. After incubation, the samples were centrifuged at 20,000*g* for 20 minutes at 4°C, and the supernatant was collected in a new tube. The protein concentration in the supernatant was measured by the BCA protein assay kit (Sigma-Aldrich, BCA1-1KT).

Equal amounts of protein (20–50 μg) were separated by 10%–15% SDS-PAGE. Following electrophoresis, the proteins were transferred onto PVDF membranes (Merck Millipore, IPVH00010). Prior to antibody incubation, the membranes were blocked with blocking solution (TBS with 0.1% Tween 20 [TBST] supplemented with 5% nonfat dried milk) for 1 hour at room temperature to reduce nonspecific binding of antibodies. For primary antibody incubation, the appropriate antibodies (see Table 3) were diluted in antibody buffer (2.5% BSA in TBST and 0.02% sodium azide) and the membranes were incubated overnight at 4°C. After washing with TBST, the membranes were incubated with horseradish peroxidase–conjugated anti-rabbit (Abcam, ab97200) or polyvalent anti-mouse (Danyel Biotech, NXA931) immunoglobulin secondary antibody for 1 hour at room temperature. Antibody detection was performed using the ECL Plus chemiluminescence Western Blot kit (Advansta, K-12042-D20).

### Quantification of mRNA by quantitative RT-PCR.

RNA was isolated from snap-frozen testes using TRIzol (Invitrogen, 15596-018) following the manufacturer’s protocol. To eliminate genomic DNA contamination, a DNase digestion step was performed using the PerfeCTa DNase I kit (Quanta Bioscience, 95150) according to the manufacturer’s instructions. Subsequently, 1 μg of RNA was reverse transcribed to cDNA using the qScript cDNA synthesis kit (Quanta Bioscience, 95048) as per the manufacturer’s protocol. Quantitative RT-PCR was carried out on a QuantStudio 12K Flex machine (Applied Biosystems) using PerfeCTa SYBR Green Super Mix (Quantabio, 95074-012-4). The primers utilized in this study are listed in Table 4. The relative level of each mRNA was normalized to *Rplp0* and *18S* rRNA (for tissue) as a reference, and the comparative Ct (ΔΔCt) method was employed for the data analysis.

### DNA extraction from FFPE tissue.

DNA was extracted from FFPE testes to quantify total bacterial load 7 days after infection. A total of 8 sections (5 μm thick) were collected in 2 mL tubes. DNA extraction was performed using the QIAamp DNA FFPE Tissue Kit (Qiagen, 56404) following the manufacturer’s instructions. As demonstrated in previous studies (35, 55–57), the isolated DNA (*n* = 5) was used for quantitative RT-PCR analysis targeting the bacterial marker gene *PapC* (primer sequence in Table 4). The relative abundance of each mRNA was normalized to *Actin* as a reference, and the ΔΔCt method was employed for data analysis.

### Isolation of bone marrow cells.

BMDMs were generated following the protocol described in Haag and Murthy (58), with slight modifications. Briefly, bone marrow cells were obtained by flushing the femurs and tibias from C57BL/6J mice (WT, *Irp1^–/–^*, and *Irp2^–/–^*). After RBC lysis, the cells were plated in complete DMEM supplemented with 20% FCS (Biological Industries), 30% CCL1 cell–conditioned medium (L929 cells were a gift from Jerry Kaplan, University of Utah, Salt Lake City, Utah, USA), 1% L-glutamine, and 1% penicillin-streptomycin. The cells were incubated for 6 days at 37°C, 5 % CO_2_, and 6% O_2_. On day 6, fully differentiated macrophages were harvested. On the day of the experiment, cells were stimulated with 200 ng/mL LPS (Sigma-Aldrich, L4391) or without LPS in a time-dependent fashion. Extracted proteins from BMDMs were for Western blot analysis.

### Statistics.

Statistical analyses were performed using GraphPad Prism software (version 8.0). All data were analyzed for normal distribution before performing statistical tests. To check for normal distribution, data were first transformed to log values. Values were used to create QQ plots and to perform Kolmogorov-Smirnov and Shapiro-Wilk tests. Two-way analysis of variance (ANOVA) followed by Tukey’s multiple-comparison test was used (in Figures 1–5 and Supplemental Figure 3) to evaluate the responses of 2 factors, i.e., UPEC infection compared to sham and IRP1/2 deletion compared to WT. For quantitative PCR (Figures 1 and 5) and Western blotting (Figure 2 and Supplemental Figure 4), all data were normalized to WT sham. Therefore, Tukey′s multiple-comparison test was performed on these data. The noninfected samples were statistically analyzed using the Kruskal-Wallis test followed by Dunn′s multiple-comparison test (Supplemental Figure 1). A *P* value of less than 0.05 was considered statistically significant: *P < 0.05, **P < 0.01, ***P < 0.001.

### Study approval.

Animal experiments were conducted according to ethical approval from the Technion Animal Ethics Committee, Haifa, Israel (IL-135-09-19) and the committee on animal care of the Justus-Liebig-University Giessen (M_819).

### Data availability.

All data needed to evaluate the conclusions in the paper are present in the paper or the Supporting Data Values file.

## Author contributions

NG and AH are co–first authors and contributed to the performance, analysis, validation, and supervision of the in vivo experiments as well as writing the manuscript. NG was responsible for the establishment of the animal model, which is why his name appears first. BAB performed the BMDM experiments. SB and NGR provided intellectual support to the concept and contributed to the editing and review of the manuscript. EGMH and AM were involved in the conceptualization of the study, supervision, acquisition of funding, and contributed to the writing and editing of the manuscript.

## Supplementary Material

Supplemental data

Unedited blot and gel images

Supporting data values

## Figures and Tables

**Table 4:**
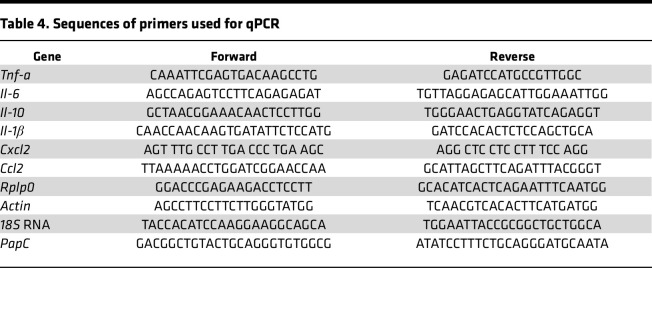
Sequences of primers used for qPCR

**Table 3:**
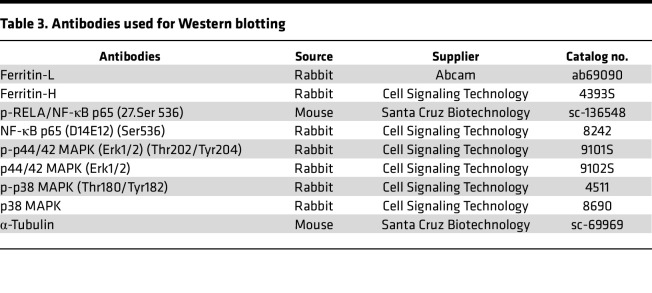
Antibodies used for Western blotting

**Table 1:**
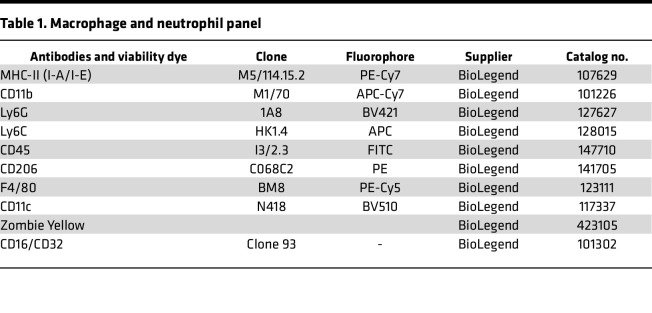
Macrophage and neutrophil panel

**Table 2:**
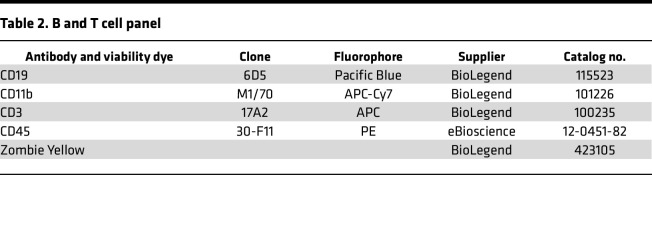
B and T cell panel

## Version 1 02/01/2024

In-Press Preview

## Version 2 03/08/2024

Electronic publication
