# Supplementary material for: Iron regulatory proteins 1 and 2 have opposing roles in regulating inflammation in bacterial orchitis
Source: JCI Insight. 2024 Feb 1;9(5):e175845. doi: 10.1172/jci.insight.175845 (PMC11143929; doi:10.1172/jci.insight.175845)
Supplement: Supplemental data [file jciinsight-9-175845-s043.pdf]

Supplemental Data

Iron regulatory proteins 1 and 2 have opposing roles in regulating inflammation in bacterial orchitis

Niraj Ghatpande[1*], Aileen Harrer[2*], Bar Azoulay-Botzer[1], Noga Guttmann-Raviv[1], Sudhanshu Bhushan[2], Andreas Meinhardt[2#], Esther G. Meyron-Holtz[1#]

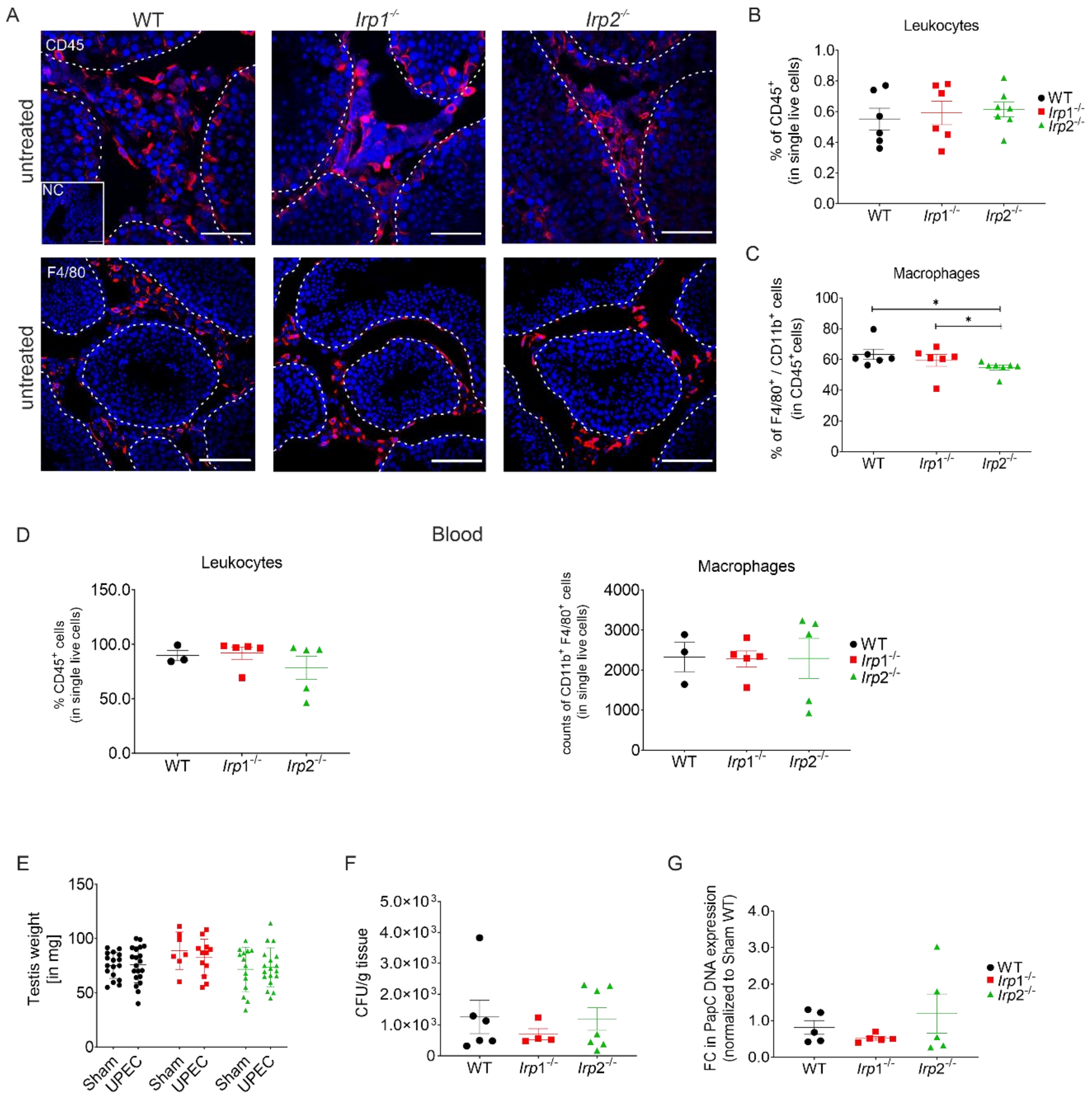

Supplementary Figure S1 (A) Immunofluorescence staining of cryosections and (B, C) flow cytometry analysis of untreated testis samples of all three genotypes (WT, *Irp1*$^{-/-}$, *Irp2*$^{-/-}$) were conducted using CD45 and CD11b and F4/80 markers to identify changes in total leukocytes and macrophages. Representative immunofluorescence images of total leukocytes (CD45) and macrophages (F4/80) are provided (N=3-6, scale bar=100µm). Statistical analysis was performed using the Kruskal-Wallis test followed by Dunn´s multiple comparisons. Mean ± SEM (D) Analysis of circulating immune cells was performed using flow cytometry on blood samples collected from WT, *Irp1*$^{-/-}$ and *Irp2*$^{-/-}$ mice (N=3-5). Representative graphs of total leukocytes (CD45^{+}; in 2x10^{5} single live cells) and macrophages (F4/80^{+} CD11b^{+}; in 2x10^{5} single live cells) are shown. (F) Testis weights were recorded after 7 days of infection, (F; G) and the bacterial load in testis were quantified using (F) CFU and (G) PapC qPCR analysis. A summary of three independent experiments is provided, with N= 4–7 for CFUs and N=5 for PapC qPCR per group.

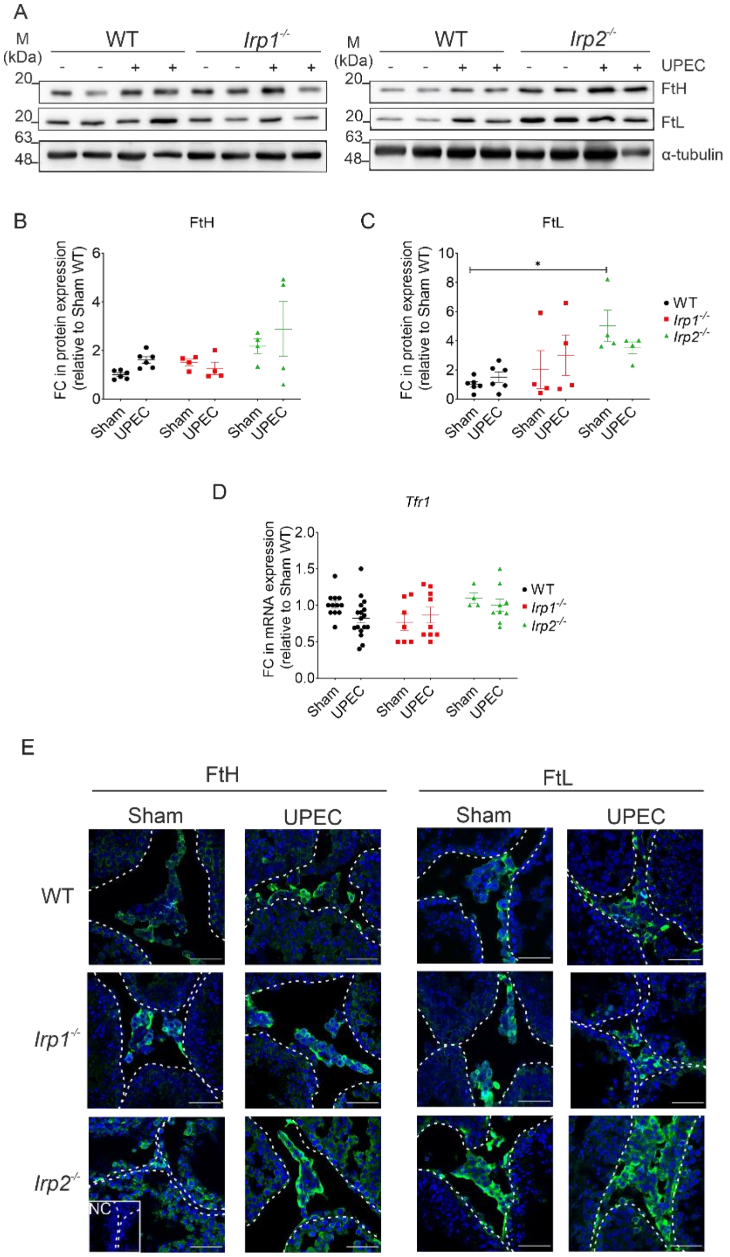

Supplementary Figure S2. (A, B) Protein levels of ferritin-H (FtH) and ferritin-L (FtL) in UPEC-infected WT, *Irp1*$^{-/-}$ and *Irp2*$^{-/-}$ testis were analyzed by Western blot. The band intensities of FtH (B) and FtL (C) were quantified using ImageJ and normalized to α-tubulin. Representative images are shown. Significant differences are indicated (**P*<0.05, ** *P*<0.01; Mean ± SEM), N=4-6 independent samples. (D) Quantitative RT-PCR analysis of transferrin receptor (*Tfr1*) in UPEC-infected testis (N= 6-15 mice per group). Mean ± SEM (E) Immunofluorescence staining was performed to investigate the changes in the localization of FtH and FtL in UPEC-infected testes. Cryosections of all three genotypes were stained for FtH (green), FtL (green) and DAPI (blue). Representative images are shown. Scale bar = 50 µm, N=5. FC=Fold change

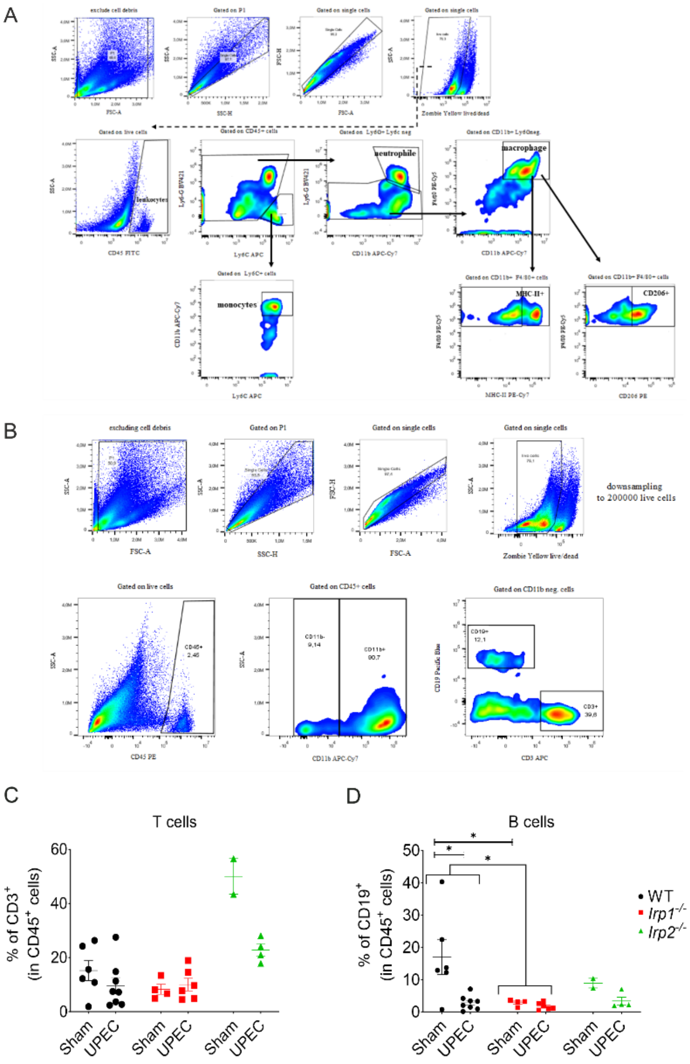

Supplementary Figure S3: (A, B) Gating strategy for the macrophage and neutrophil antibody panel in infected testis samples. Single live cells were initially gated for CD45 to select total leukocytes. From the $CD45^+$ population, monocytes were further gated using Ly6G and Ly6C expression. The gate of $Ly6G^+$ and Ly6C- cells was then used to identify granulocytes using CD11b and Ly6G expression. After excluding the monocytes and granulocytes, macrophages were gated ($CD11b^+Ly6G^-$). Finally, macrophages and their subpopulations were identified using the following markers.: $CD11b^+F4/80^+$, $F4/80^+MHC\text{-}II^+$, and $F4/80^+CD206^+$. (B-D) Gating strategy and respective graphs of B and T cells are presented. Single live cells were initially gated for CD45 followed by gating on CD11b alone (B) with subsequent selection of the $CD11b^-$ population for gating on CD19 (B cells) and CD3 (T cells) expression, respectively. The graphs depict the percentages of B cells (C) and T cells (D) within the $CD45^+$ population. Data were obtained from infected testis samples and represent the summary of three independent experiments (each N= 2–3 per group). Statistical significance was determined using two-way ANOVA with Tukey multiple comparison (N=6; *P<0.05, ** P<0.01). Mean ± SEM

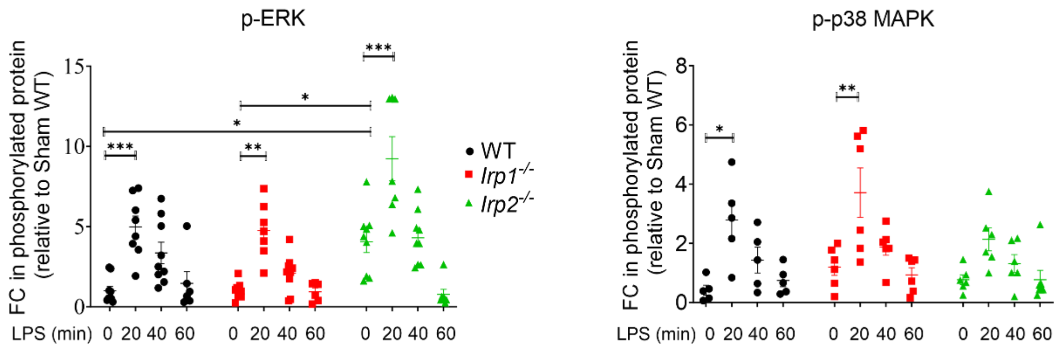

Supplementary Figure S4: Bone marrow-derived macrophages (BMDM) from WT, *Irp1*$^{-/-}$ and *Irp2*$^{-/-}$ mice were isolated and stimulated with 200 ng/ml lipopolysaccharide (LPS) for different time points (20 and 60 min) under specific oxygen conditions (6% O_2 at 37°C). Protein levels of indicated signalling molecules were analyzed by Western blot. Representative images are shown. Quantitative analysis of the band intensities of p-ERK and p-P38 were performed using ImageJ and normalized to the corresponding loading control. Statistical analysis revealed significant differences (*P<0.05) between the genotypes and time points. Mean ± SEM, FC= Fold change
