# Supplementary material for: Iron regulatory proteins 1 and 2 have opposing roles in regulating inflammation in bacterial orchitis
Source: JCI Insight. 2024 Feb 1;9(5):e175845. doi: 10.1172/jci.insight.175845 (PMC11143929; doi:10.1172/jci.insight.175845)

Full unedited gels for Figure 2A. Left panel

TLR4 — Irp1-/- | WT

\+ + - - + + - - UPEC

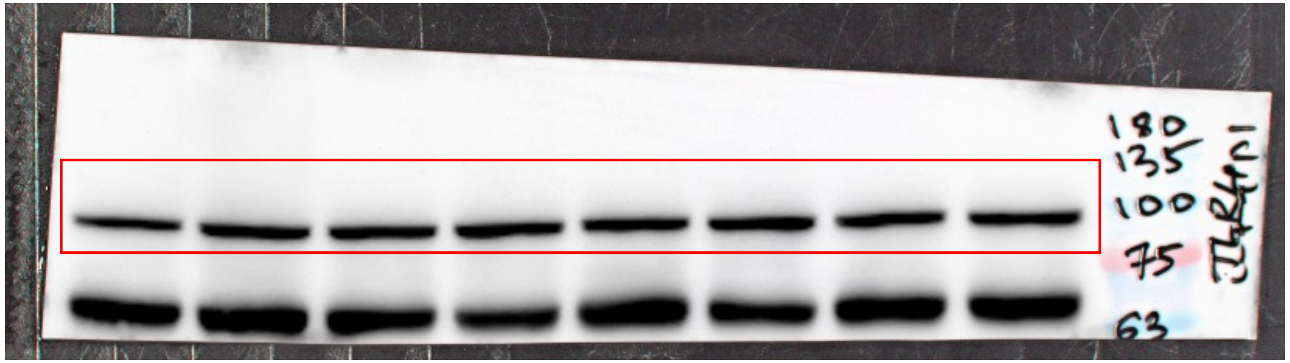

p-ERK

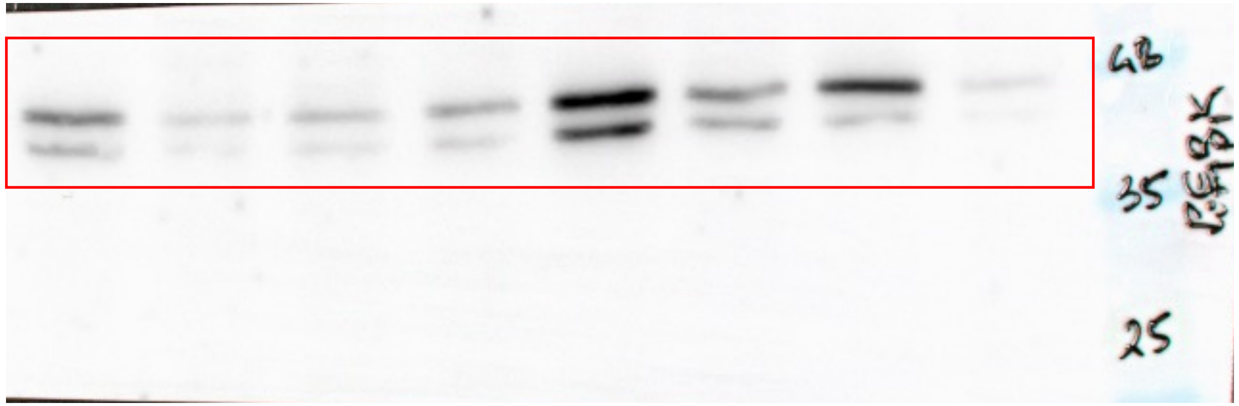

ERK — Irp1-/- | WT

\+ + - - + + - - UPEC

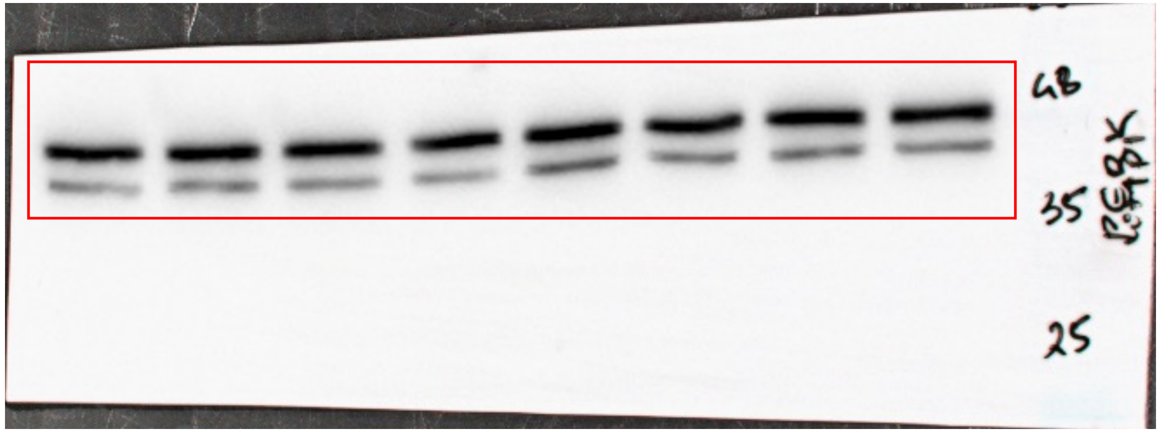

Full unedited gels for Figure 2A. Left panel

p-P38 Irp1-/- WT

\+ + - - + + - - UPEC

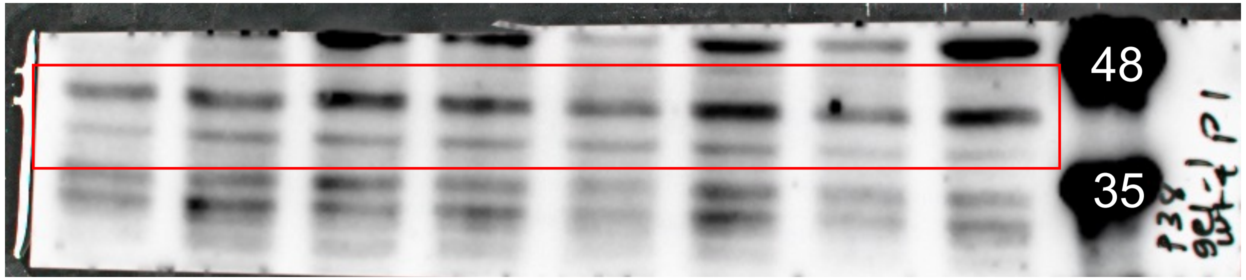

P38

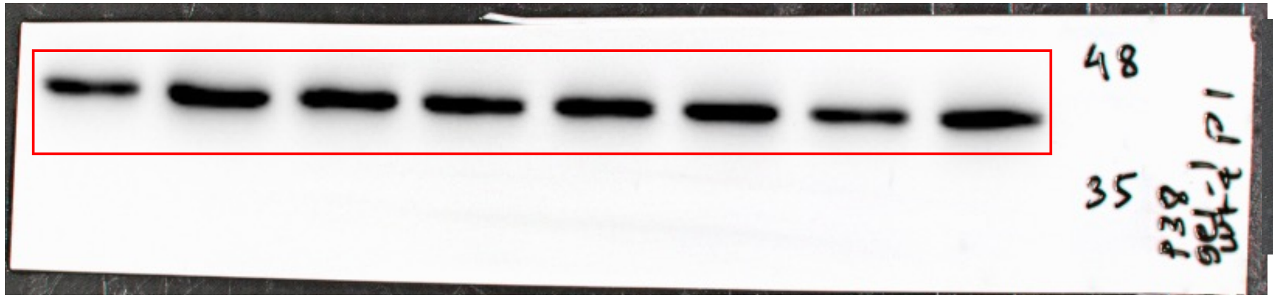

p-P-65 Irp1-/- WT

\+ + - - + + - - UPEC

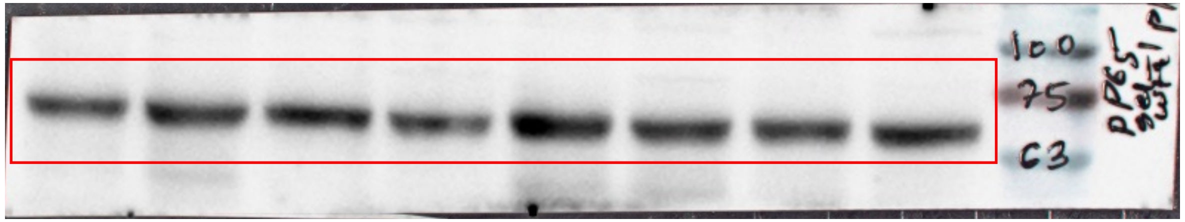

P-65

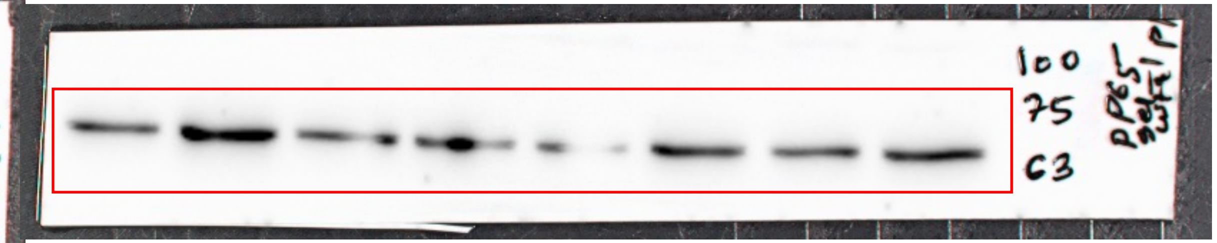

Full unedited gels for Figure 2A. Right panel

TLR4

Irp2-/- WT

\+ + - - + + - - UPEC

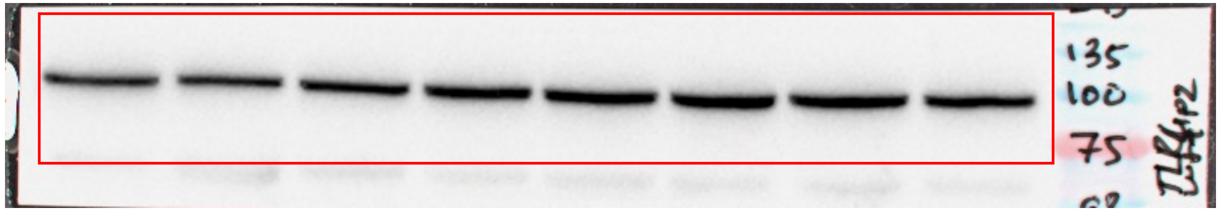

p-ERK

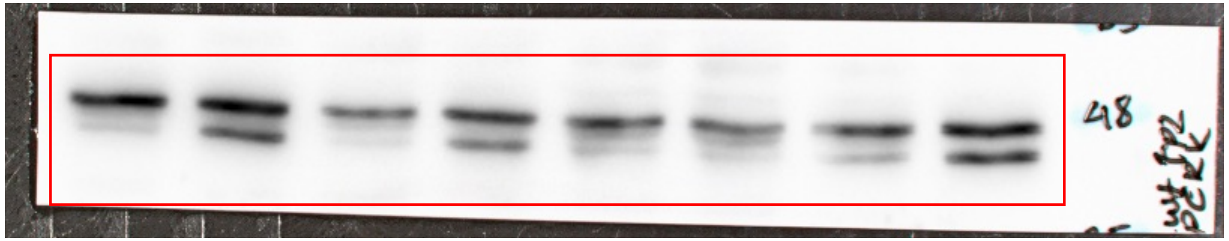

ERK

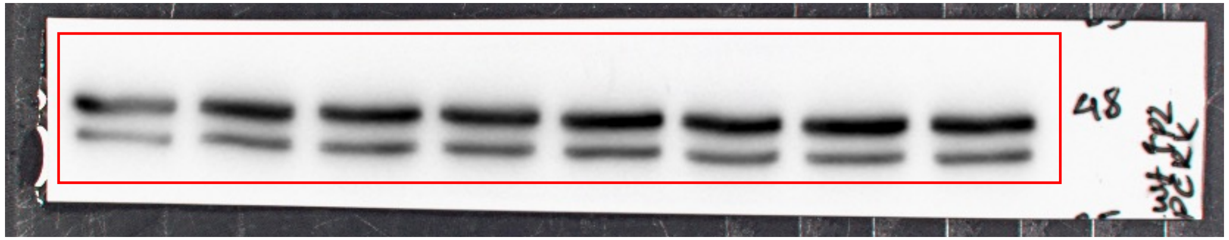

p-P38

Irp2-/- WT

\+ + - - + + - - UPEC

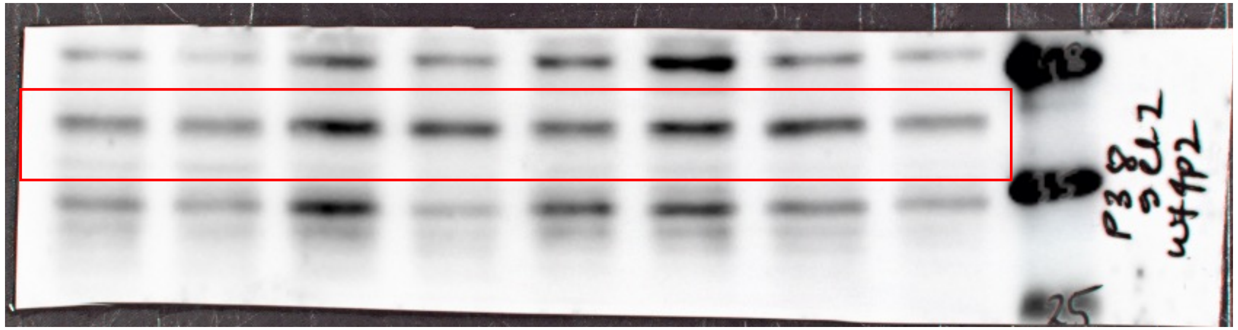

P38

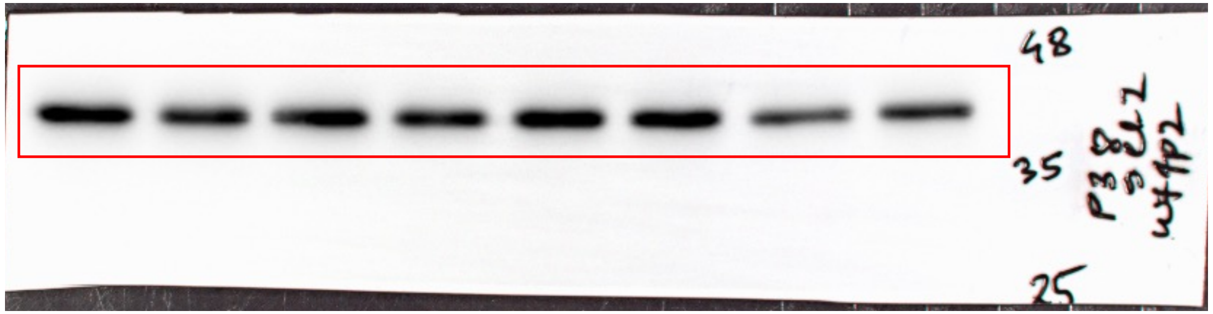

Full unedited gels for Figure 2A. Right panel

p-P-65

Irp2-/- WT

+ + - - + + - - UPEC

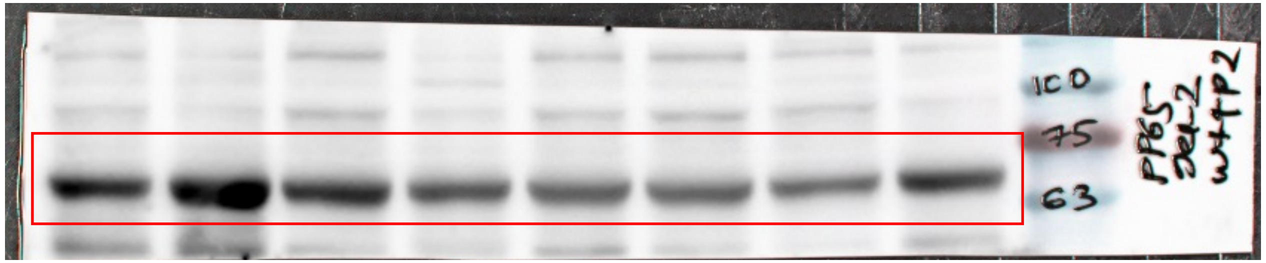

P-65

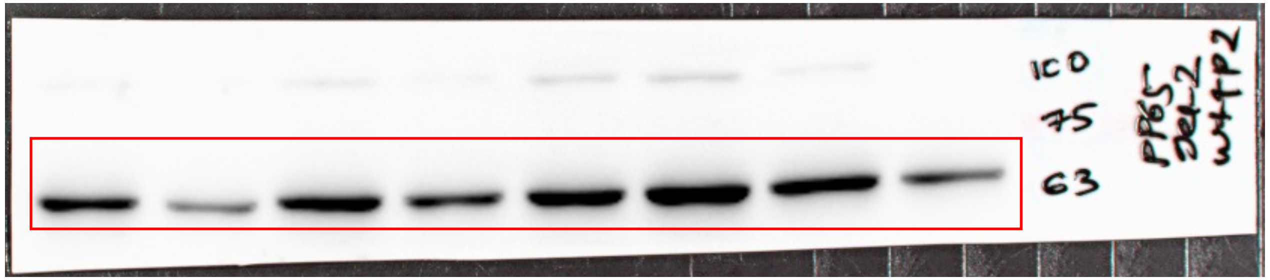

Full unedited gels for Figure 2C.

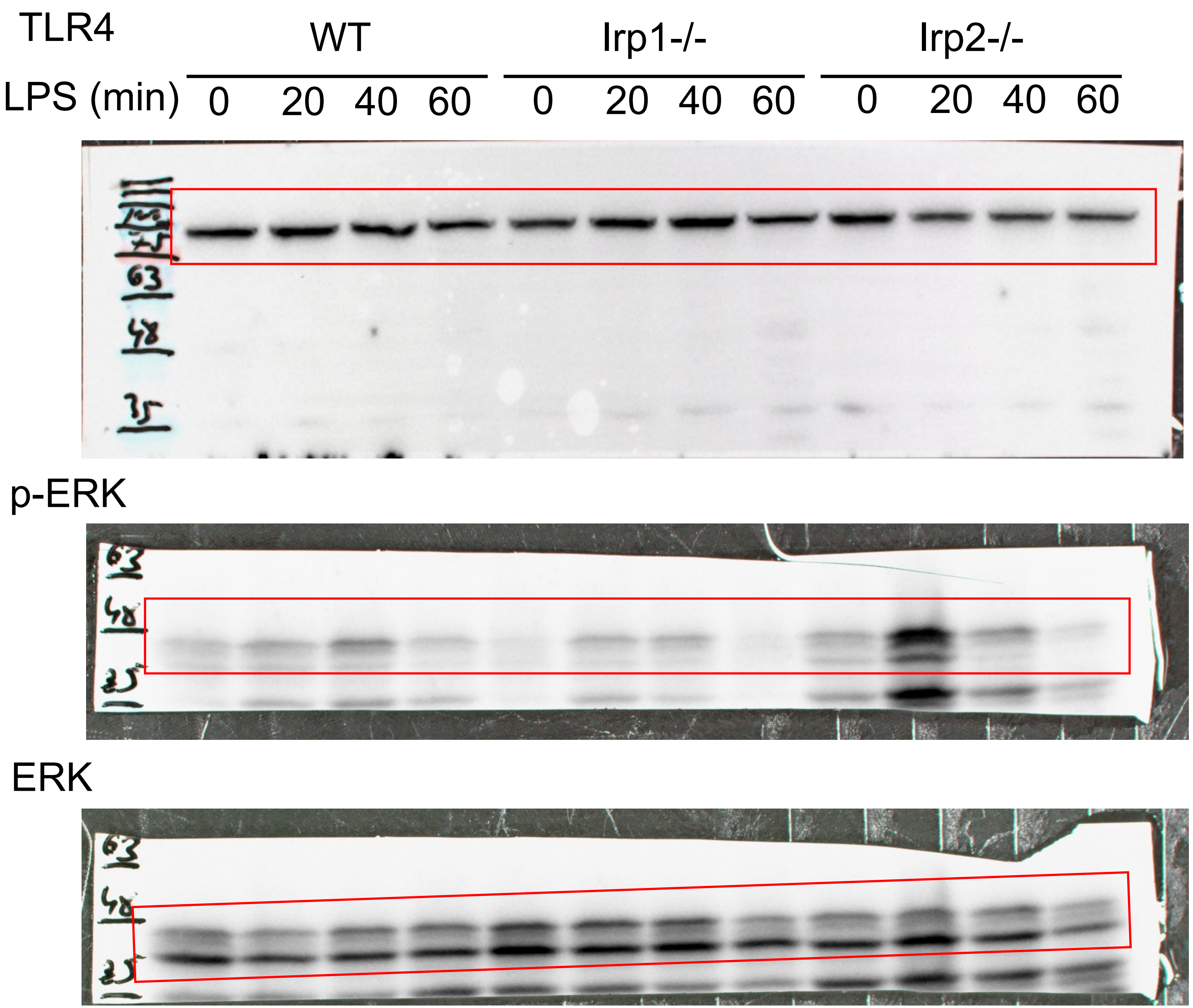

Full unedited gels for Figure 2C.

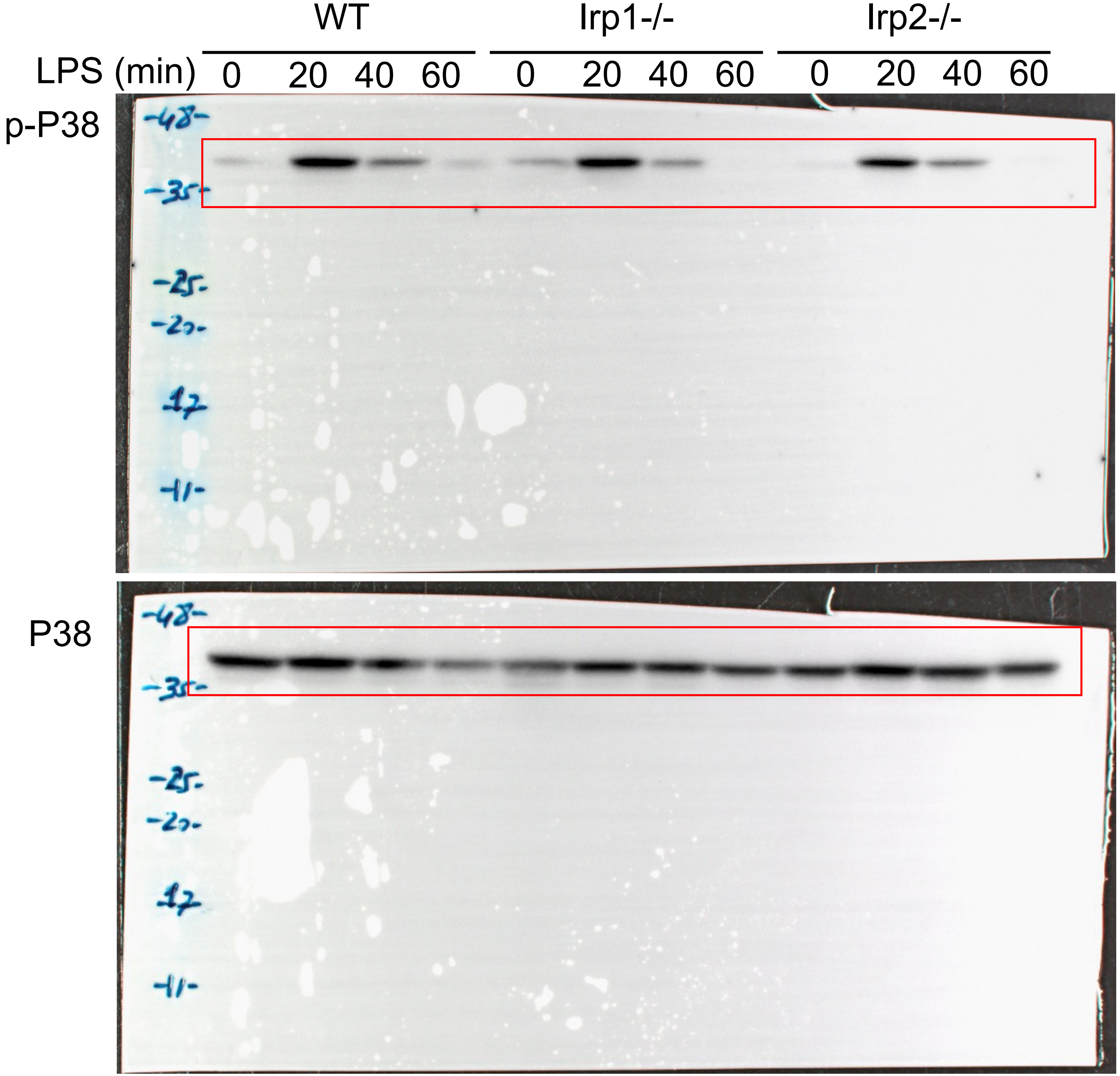

Full unedited gels for Supp. Figure 2A. Left panel

FtH Irp1-/- WT

\+ + - - + + - - UPEC

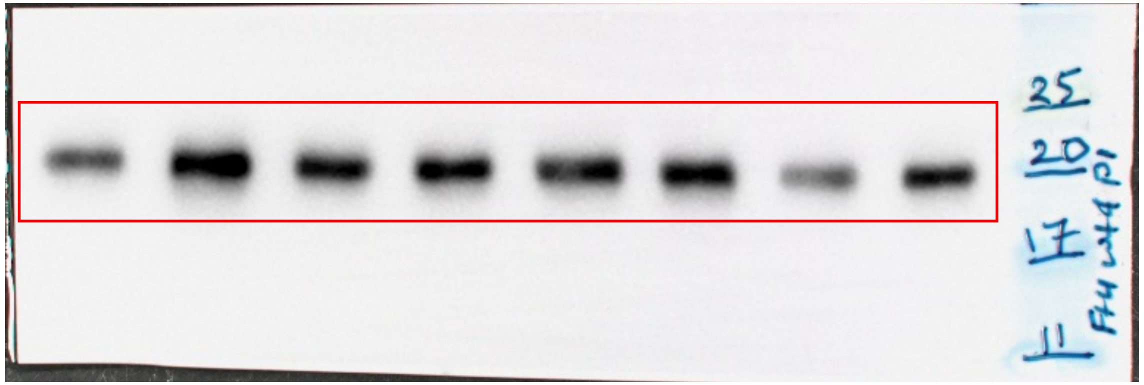

FtL

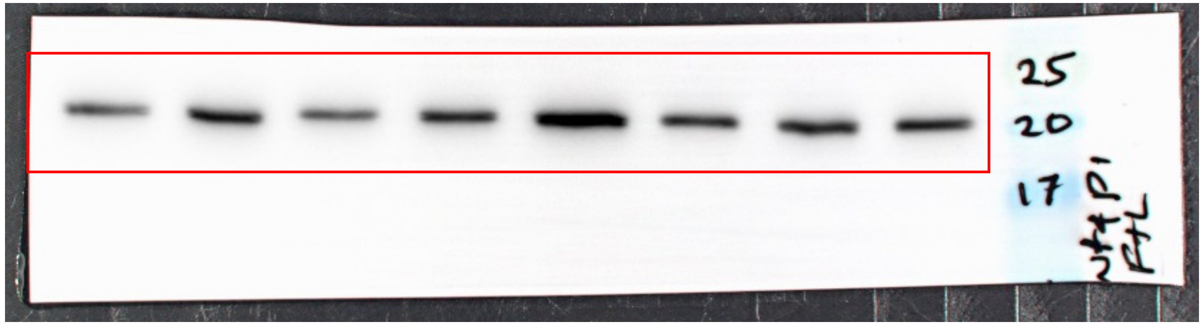

α-Tubulin

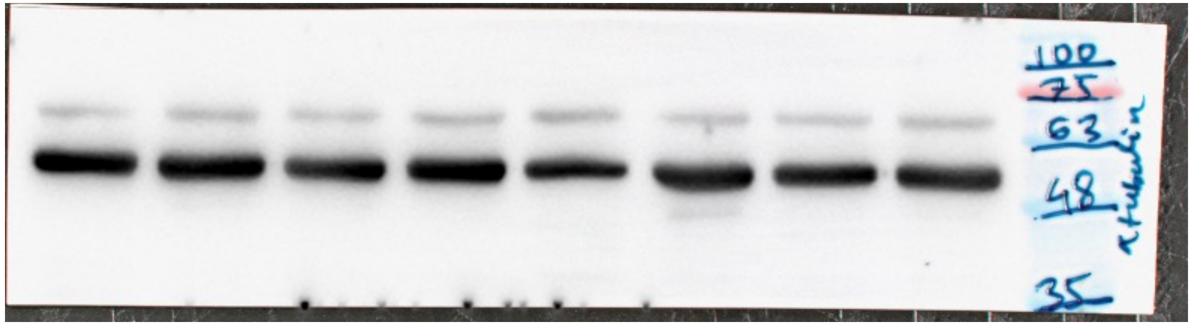

Full unedited gels for Supp. Figure 2A. Right panel

FtH Irp2-/- WT

+ + - - + + - - UPEC

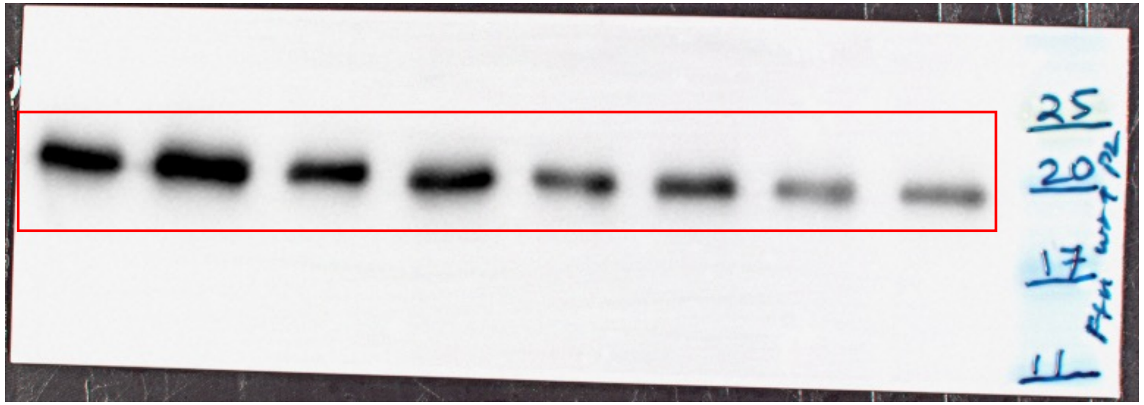

FtL

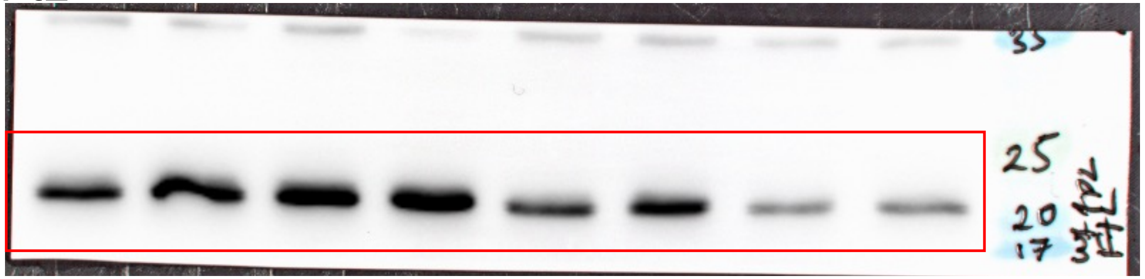

α-Tubulin

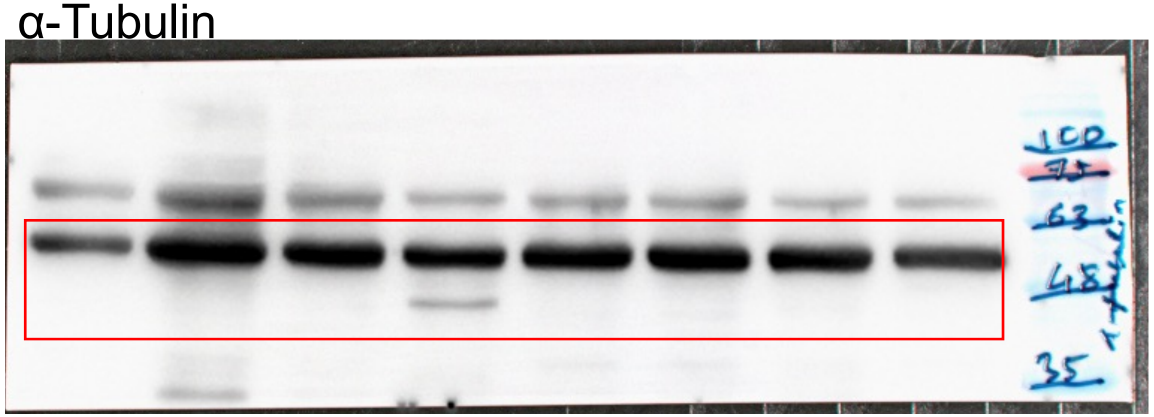

Supplement: Unedited blot and gel images [file jciinsight-9-175845-s044.pdf]
